# An Experimental Comparison of the Impact of ‘Warning’ and ‘Health Star Rating’ FoP Labels on Adolescents’ Choice of Breakfast Cereals in New Zealand

**DOI:** 10.3390/nu12061545

**Published:** 2020-05-26

**Authors:** Robert Hamlin, Benjamin Hamlin

**Affiliations:** 1 Department of Marketing, University of Otago, PO Box 56, Dunedin 9054, New Zealand; 2 Taieri College; 3 Green Street, Mosgiel 9024, New Zealand; raphamlin@xtra.co.nz

**Keywords:** FoP, front of pack, nutritional, label, nutrition, obesity, traffic light label, TLL

## Abstract

This research investigated the performance of the red, octagonal Vienna Convention traffic ‘STOP’ sign as a front of pack (FoP) warning nutritional label. While the Vienna Convention traffic light system is an established FoP label, the potential of the ‘STOP’ sign in the role has not been investigated. The performance of the ‘STOP’ label was compared with that of a single star (low nutritional value) Australasian Health Star Rating (HSR) label using a fractionally replicated Latin square design. The labels were presented on choice diads of cold breakfast cereal packets. The sample of 240 adolescents aged 16–18 was drawn from a secondary school in the South Island of New Zealand. A large and significant main effect was observed at the *p* < 0.01 level for the difference between the ’STOP’ sign and the control condition (no nutritional FoP label), and at *p* < 0.05 for the difference between the HSR and the ‘STOP’ label. There was no significant difference between the HSR FoP and the control condition. A significant non-additivity (interaction) (*p* < 0.01) was also observed via the fractional replication. The results indicate that the Vienna Convention ‘STOP’ sign is worthy of further research with regard to its potential as an FoP nutritional label.

## 1. Introduction

When front of pack (FoP) labels first appeared, the intent of their backers was purely to inform food consumers, resulting in systems such as the recommended daily intake (RDI). However, later label formats sought not only to inform consumers, but to persuade them via a degree of evaluative content [1]. Matters have now advanced to the point that Jones (2019) based their analysis on 31 government endorsed systems, with a bewildering variety of formats that range from the purely informative to the fully evaluative [2]. This complexity increases the diversity of regulatory, calibration and domestic/international legal issues that jurisdictions have to deal with [3]. 

The degree to which this diversity in FoP format increases regulatory ‘load’ in a range of areas was noted by a recent WHO report [4], and one of the two major recommendations was to standardize FoP formats around the world. Standardization would allow resources to be more efficiently concentrated behind a smaller number of FoP formats. A smaller standard population of FoP formats would also present a smaller target for those who would wish to impede the deployment of these systems by whatever means [3]. 

Standardization must mean elimination on a large scale, and ideally on the basis of the absolute and comparative performance of the many FoP label formats in influencing consumer behavior before any significant regulatory investment is made, and especially before any system is ‘bedded in’ by compulsion. The necessary yardstick of performance is not hard to identify. An FoP label is only effective if it significantly alters consumer behavior towards the food products that carry it, and affects it in the manner intended. It is perfectly possible for an FoP to succeed on the former criterion and fail in the latter [5]. 

Thus, reliable comparative testing is required. While this objective is clear cut, the methods required to achieve it are challenging, especially in relative terms. The ideal test would be a large-scale set of controlled experiments using real stores, real products and real consumer purchases. This is perfectly technically feasible, but would require a degree of funding and industry cooperation at all levels that would almost certainly require direct state intervention to deliver. 

It is noteworthy that while some reports recommend compulsory enforcement of some FoP labels [3], none have so far recommended the eminently logical prior step of requiring compulsory industry cooperation to properly test whether the FoP label in question justifies its place on the package in the first place.

In the absence of such definitive tests, smaller scale experimental exercises to investigate relative and absolute FoP performance along the consumer choice yardstick must, for the time being, suffice. This article describes a test of this nature that examines the relative performance of the Australasian Health Star Rating System, and a version of the increasingly popular ‘warning‘ labels. As the generation of ‘clean’ datasets that can support the accurate measurement of consumer response to package cues is a tricky process [6], the means by which this test was conducted is discussed in considerable detail. 

### 1.1. Background 

**Figure 1:**
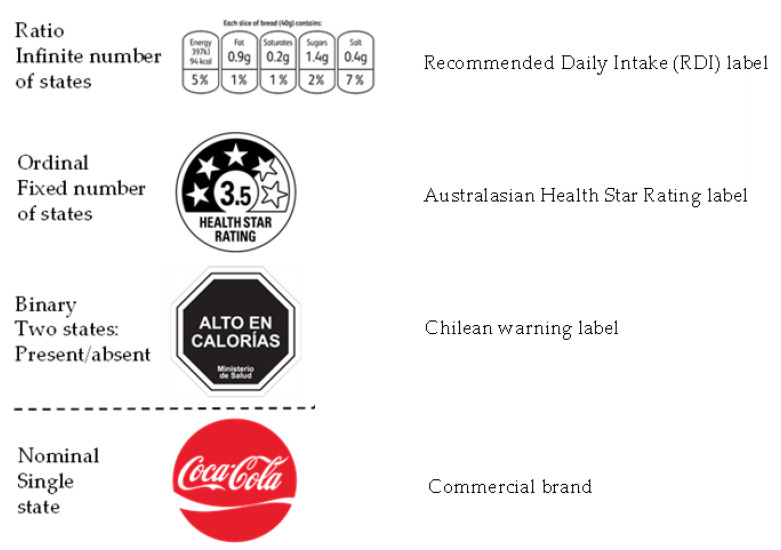
A classification of cues that are encountered on food products at the point of sale. Front of pack (FoP) labels display a wide variety of formats, but only represent a small proportion of the total cues encountered. The vast majority of package cues are nominal and proprietary commercial brand related logos and images, but also include non-proprietary health claims etc. A nominal cue is a recognizable ‘shape’, which may be a word, a design or a combination of the two that has a specific non-dimensional meaning to its target, e.g., the Coca-Cola® brand). Some widely deployed generic (ownerless) cues, e.g., ‘lite’/’organic’, may display both binary and nominal characteristics.

The recent emergence of front of pack (FoP) nutritional ‘warning’ labels, most notably the system developed in Chile based upon a battery of black octagons, has generated considerable research interest [7,8,9,10,11,12,13,14] (Figure 1). This system has been the subject of several studies with regard to its performance relative to other established FoP systems over the last four years [15,16,17]. The outcomes of the research indicate that warning label systems may be more effective than alternative FoP systems with regard to influencing consumer evaluation and purchase of food products [18,19,20,21].

The individual black octagons of the Chilean warning label system form part of a family of FoP labels known as ‘binary cues’ (Figure 1) [1]. Cues of this type communicate the status of a product by their presence or absence (their two ‘binary’ states). This is in contrast to the other established FoP nutritional label systems, which are either ‘ordinal’ cues, on which the label is always present and expresses multiple discrete nutritional states on a positive negative continuum (e.g., traffic light label/HSR) or ‘ratio’ cues, in which an infinite number of nutritional states on a positive/negative continuum may be expressed (e.g., percentage daily intake (PDI) label). While binary format FoP nutrition labels have been deployed for some time before the Chilean system, they have been endorsements rather than warnings (e.g., the ‘Heart Foundation Tick’ and the ‘Swedish Keyhole’ [22,23]. 

**Figure 2:**
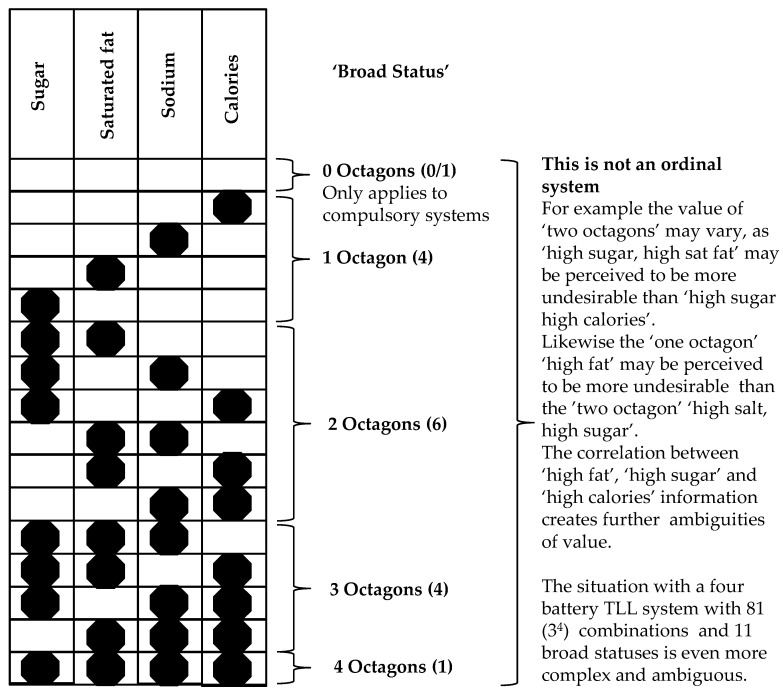
The geometric increase in complexity when multiple labels are used. The Chilean octagon system based upon four binary octagonal labels.

Binary FoP labels are the closest approach to the nominal cue format that forms the basis of commercial food branding and marketing communication practice [24]. However, it should be noted that using multiple warning labels destroys this binary status, and introduces a level of complexity that increases exponentially with the number of labels used. The four octagon Chilean system for example has 16 possible combinations arranged in five broad statuses with zero to four octagons. However, as each ‘state’ comprises multiple combinations of octagons of indeterminate relative value, the system cannot be described as ordinal (Figure 2).

The regulatory uptake of FoP warning labels has been swift and decisive. In addition to Chile, compulsory FoP systems based on this approach were introduced in Peru in 2018 [25,26], and they are approved for introduction in Israel in 2020 [27,28] and are under active development in Canada, Mexico and Uruguay [9,29,30,31]. The sharp move towards compulsion is noteworthy, and is in contrast to the established FoP formats, which remain voluntary in the areas where they have been deployed, even when these labels have been deployed in those areas for a long period of time.

The rapid move to deploy compulsory FoP systems based upon warning labels may be premature given the still rapidly developing body of research evidence that is currently available to decision makers [10]. This lack of evidence not only applies to the efficiency of the warning label format in general, but also to exactly which warning label design/system is the most efficacious format.

**Figure 3:**
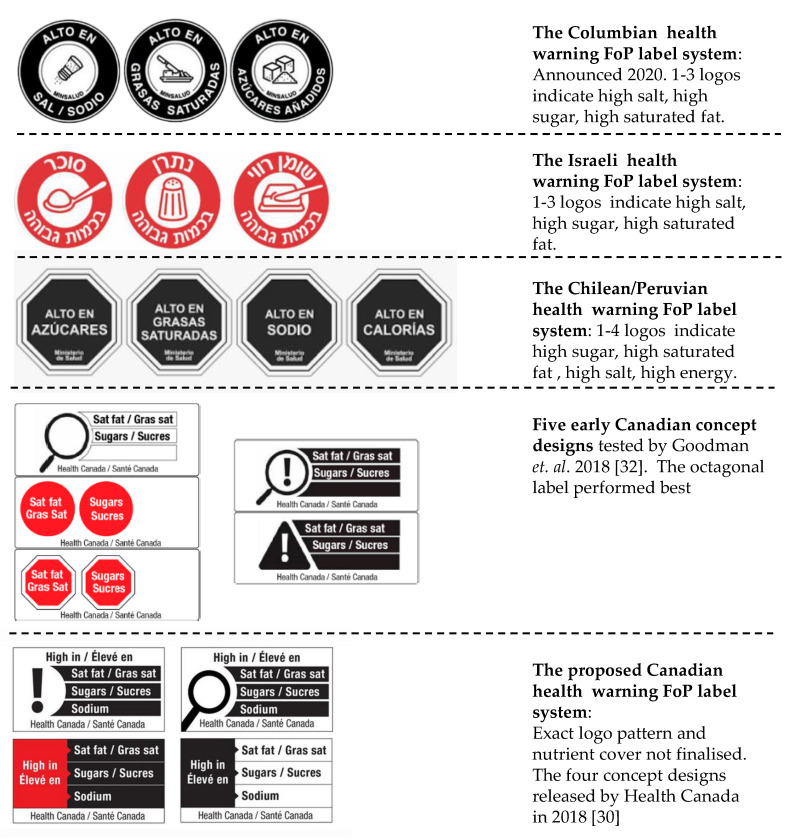
A sample of the warning label formats that are deployed or under development worldwide.

Despite the very high visibility of the Chilean black octagon design, there are a very wide variety of alternative FoP warning designs that are either already deployed or under active consideration worldwide. Figure 3 shows a sample of the various formats for existing and proposed FoP warning label systems around the World. The very wide range of colors, formats, information payload and arrangements shown in this figure indicates that there is considerable opportunity for researchers to usefully contribute to informing policy by testing consumer response to them, both absolutely and relative to one another. 

### 1.2. Food Consumer Education and the Use of Established International Warning Sign Formats

One of the biggest problems facing any food marketer is how to ‘educate’ their consumers about how to respond to any logo or symbol. Food purchase is dominated by passive and subconscious ‘low involvement’ consumer learning and decision processes [32,33,34]. Educating consumers about the meaning of any particular logo to the point where it will significantly influence these processes is both a time consuming and enormously expensive task—as the enormous advertising budgets and equity values of major food brand logos clearly indicate [35]. The task facing any public agency that wishes to develop an effective FoP label is exactly analogous to that of a private food marketer who wishes to create an effective brand. A nominal brand logo is merely a meaningless (and worthless) picture until that picture has been given a consistent meaning in the minds of its target market. This analogy is particularly relevant to binary format warning labels as they resemble nominal format commercial brands logos more closely than any other FoP type. The difference between brands and FoP labels is that FoP labels rarely have the supporting budget of their commercial equivalents. For this reason, it makes sense to ‘borrow’ a visual logo to which consumers have already been trained to the desired pattern of response within another application/environment.

Recent research on the warning label design options under consideration in Canada indicated that a red octagon design was the most favored among the concepts developed by Health Canada [30]. The standard VCSTOP design came a close second to the black octagon design in the Chilean development research [3]. However, this red octagon design was not favored by the Chileans and does not appear to have survived into the final quartet of designs that were publicly proposed by Health Canada as a basis for the proposed FoP warning system [29] (Figure 3). 

The red octagonal label used in both exercises has very clear similarities to the red, octagonal ‘STOP’ road traffic sign that has become a global standard method to communicate the message for motorists to stop and consider in order to avoid danger. This design standard originated with the Vienna Convention on Road Sign and Signals 1978, the international agreement that governs the design of road signs on the roads of most developed countries in the World [36]. 

As the public in Canada were already trained to this road sign in a negative/warning message, their greater understanding of and response to the octagonal shape and red color when deployed in a similar role as a food warning label is understandable [34]. While the Chilean Black Octagon format does not share the Vienna Convention STOP (VCSTOP) sign’s color, it does share its shape and layout – and a VCSTOP design was runner up in that research, and the winner in another South American research exercise [12]. As the VCSTOP logo is a tightly controlled global design standard and is consistently backed up by large budgets, formal training, constant consumer exposure and penalties for not reacting to it appropriately, this outcome is understandable. 

The opportunity to exploit consumer pre-conditioning to Vienna Convention signs also appears to have driven the initial development of the well-established ordinal traffic light FoP label system too. The initial research on the application of the system to dietary communication notes that the objective was to utilize already developed consumer subconscious responses to the Vienna Convention standard red/amber/green traffic light road signaling system [37].

### 1.3. Research Hypotheses 

Further research into the application of the VCSTOP as a ‘ready-made’ FoP warning label that could be applied to many countries with an existing basis of consumer training in the meaning of the logo thus seems to be justified. Research to immediately test the effectiveness of the VCSTOP design as an FoP warning format can be meaningfully undertaken almost anywhere, as the VCSTOP is fully developed and is already widely and consistently deployed in almost every country worldwide. 

The research undertaken by Goodman et al. [30] was developmental. It investigated consumers’ self-reported opinions about the various designs proposed by Health Canada. It did not investigate if any of the individual designs had any impact upon the targeted consumer behavior—reducing purchases of unhealthy foods. This research developed from that base by directly investigating the VCSTOP’s performance with regard to influencing unprompted consumer choice of food products in New Zealand. The objective was to test the performance of the VCSTOP both absolutely against a no-label control condition, and relatively against the most negative setting of the locally established Australasian Health Star Rating (HSR) system that the researchers had observed in the cold cereals category.

The HSR system has been deployed in New Zealand for nearly five years, with considerable government and business support aimed at creating community awareness and association of the label and its message [38]. It thus represented an appropriate benchmark for the VCSTOP format. The development and deployment of the HSR has also been addressed by a considerable body of published research. The results of this research have been inconclusive with regards to its capacity to influence consumer choice [2,38,39], although some impact upon producer behavior has been noted [40]. The system has attracted criticism with regard to the algorithm that drives its 1-5-star nutritional rating, which is particularly insensitive to high sugar levels [41]. A formal five year review in the HSR was published in 2019, which recommended a continuation of the system, but not a move to compulsion [42], which makes a comparative examination of its performance against a warning label relevant. Recent Australian research using a complex and novel warning label indicated that even this unknown format outperformed the established HSR in influencing consumer choice [43]. However, on the basis of the more developed Canadian warning label research [30], the researchers chose to use the VCSTOP design in this research.

Cold breakfast cereals were selected as the category for the study as many products within the category have a high sugar content [44], and the category was the setting for a recent marketing war between its three major participants (Sanitarium, Kellogg’s and Nestle) on the basis of HSR ratings, which led to a very high level of advertising support in all commercial media for the HSR within the category [38]. This, connected with the extensive adverse comments in the general media noting that many of these high HSR scoring cereals were actually very high in sugar, created a very high level of awareness of the HSR with regard to sugar and this type of product [45]. 

While the researchers intended to test the label with a variety of consumer groups, an opportunity arose in late 2018 to conduct a research exercise of this type with a large sample of New Zealand Adolescents aged 16–18. 

New Zealand, like Chile, has an obesity problem [46]. However, New Zealand has some very specific cultural issues with regard to obesity. Along with many other English-speaking countries, dietary and behavioral patterns have changed in recent decades, and they have changed in ways that have led to increasing levels of obesity [47]. In addition, the country has a significant and increasing Polynesian population, split between Maori and Pacific Islanders (16.5% and 9%, respectively, in 2019) [48]. These dietary changes have affected these minority populations significantly more severely, with very high and rapidly increasing levels of morbid obesity and diabetes [49]. These changes, and their negative outcomes, are also establishing themselves very early in life [50]. These issues have been addressed by government policy with regard to nutritional education in schools since 2000. Nutrition education is actively delivered into all secondary schools [51,52,53]. 

The research was thus designed to test two hypotheses with regard to this young sample’s unprompted intent to consume cold breakfast cereals:

**Hypothesis** **1.** *A VCSTOP label significantly reduces unprompted intent to purchase unhealthy breakfast cereals relative to a no label (control) condition.*

**Hypothesis** **2.** *This reduction ‘ceteris paribus’ is greater than the Australasian HSR system when this is set to its minimum commonly encountered nutritional status (one star).*

## 2. Materials and Methods 

### 2.1. Research Timing and Location 

The research was conducted at a single secondary school in Southern New Zealand in October and November 2018. The researchers accessed years 11-13 of this school, generating a sample of 231 students aged between 16 and 18 years with an average age of 16.5 years. The gender ratios were: (M: 39%, F 58%, GF 3%). The secondary school is a large school with a roll of approximately 1200. The school serves a single community. The methodology was reviewed by the University of Otago Human Ethics Committee, and was approved under the ‘A’ category ethical approval system (Otago University Human Ethics Committee 18/124). 

### 2.2. Experimental Design

The research was a ‘discrete choice’, between-subjects experiment, which measured the impact of the FoP labels on the participants’ unprompted choice between cold breakfast cereal products [54]. 

**Figure 4:**
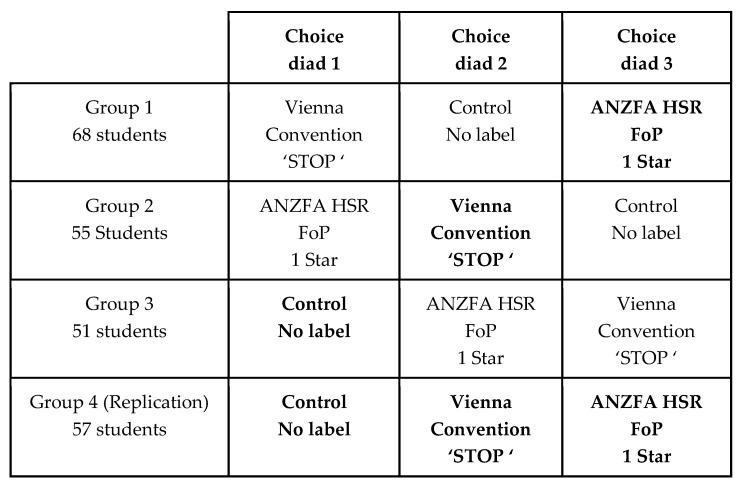
Experimental design: a 3 × 4 partially replicated Latin square by Youden and Hunter (1955) [55].

The experimental design was a 3 × 4 fractionally replicated Latin square design after Youden and Hunter (Figure 4) [55,56,57,58,59]. This design delivers a very high level of statistical efficiency, and allows for the main effects of a single independent variable of interest (the FoP label treatments) to be studied while controlling for the main effects of two other unavoidable extraneous variables. These extraneous variables were the cold cereal products on which the FoP labels were presented, and the student groups. The fractional replication (shown as bolded text in the cells in Figure 4) allowed the researchers to estimate the degree of non-additivity (interaction) between the main effects of the three variables [60]. 

### 2.3. Independent Variable 

**Figure 5:**
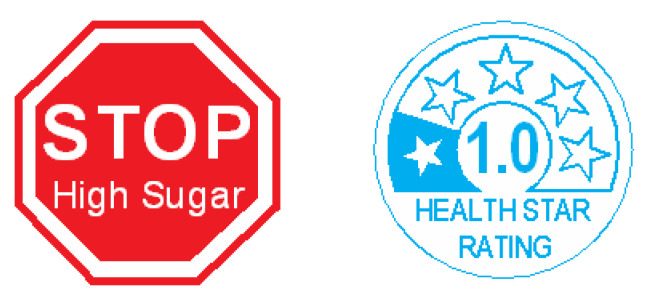
The two non-control levels of the FoP label independent variable. These were sized to occupy 3% of the prime facing of each product. The third treatment was a no label control.

The independent variable was the FoP label. The three levels of the independent variable are shown in Figure 5. They consisted of:

1) The VCSTOP label reproduced as specified by the Convention, but with ‘High Sugar’ added in a smaller font.
2) The AUSTRALASIAN HSR label set to a single star, the lowest level that has been observed in the cold breakfast cereal category in New Zealand, and without the optional PDI/RDI ‘add on’ bar. This label was thus set to act as an equivalent warning status to the VCSTOP label [61].
3) A ‘no FoP’ label control treatment.

Some consideration was given to the format of VCSTOP. The unmodified VCSTOP could not be used as it did not deliver context. Any consumer would be confused with the instruction to ‘STOP’ if no indication as to what they should stop, or why they should do so, is given as part of the label! The Chilean warning system dispenses with the red color and the word ‘STOP’ altogether, retaining only the octagonal shape and the relevant nutrient [7]. The warning label used in the recent Australian research used a complex design that included the word ‘STOP’, and some elements of the color, but not the shape [41]. The Canadian warning label research used the VCSTOP unmodified, bar the addition of ‘high’ followed by name of the nutrient below it [30]. The researchers decided to use this Canadian format as the primary independent treatment. A single VCSTOP relating to sugar was the only term used, as this is the primary nutritional issue with the cold cereal products used in this research. 

**Figure 6:**
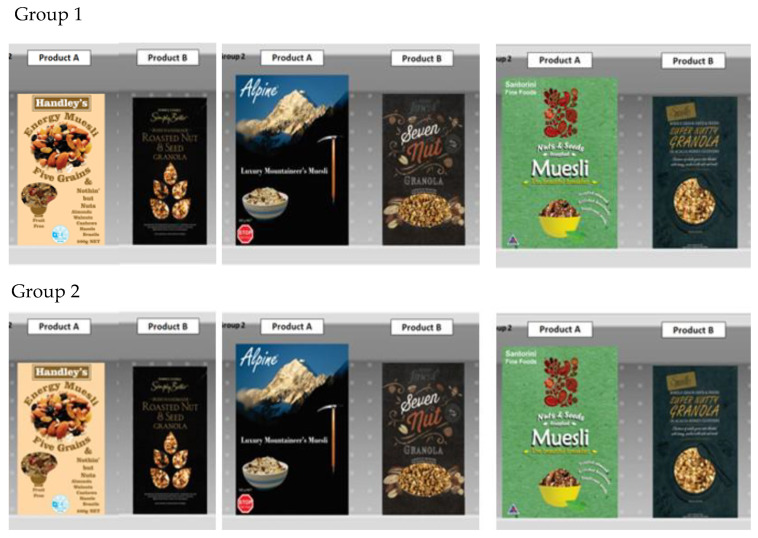
Experimental design details showing the product diads used to present the FoP labels to Groups.

The two non-control FoP label treatments were both sized to be approximately 3% of the prime facing of the packages that they were presented upon—a typical proportion of prime facing coverage found in New Zealand for the HSR roundel, where FoP label size is not regulated (Figure 6).

### 2.4. Extraneous Variables 

The first extraneous variable was the cold cereal product on which the FoP labels were presented. The FoP labels were presented on one of a pair of cold cereal products that formed a ‘choice diad’—the research participant had to choose one of these two products (Figure 6). The three choice diads used in this research were based on six ‘designed for the purpose’ muesli products, which were very similar in nature. The branding and designs were based on products that were for sale in the Irish Republic at the time, all of which were unknown to the research participants. The intent was to avoid introducing strong product/brand associations which might interact significantly with the FoP treatments. The six products were randomly assigned to the three diads. One product from each diad was selected to act as the cue vehicle, which carried the three FoP treatments, while the second product of the diad acted as a comparator and was used without any FoP labels. Figure 6 shows the three diads shown to Groups One and Two in the experimental design shown in Figure 4., along with the research instrument. The product diads remained the same for Groups Three and Four, with only the FoP labels changing in accordance with the pattern shown in Figure 4. 

The second extraneous variable was the student respondent groups. As the researchers wished to obtain an unprompted reaction to the independent variable, it was essential that the purpose of the research was concealed from them as the research was conducted. In order to represent a real choice/purchase situation, the task that the individual members of each group had to perform was to choose between the two products within each diad. If they were aware (prompted) that the purpose of the research was to investigate the FoP labels, then the subjects would interpret their task as an evaluation of the FoP labels and undertake a choice task between the label and not the products – thus making the results unrepresentative of a real product choice situation.

The Latin square design allowed the purpose of the research to be concealed if the student respondents were divided into groups and then used as an extraneous variable in a between subjects design (Figure 4). As the students never saw the same product with different levels of the independent variable, they are unable to isolate any systematic variation in the diads overall – and thus they could not ascertain the purpose of the research. Figure 6 shows this confounding process in action [6,59]. 

### 2.5. Dependent Variable

The dependent variable was derived from a ‘discrete choice’ between the two products in each of the three diads evaluated by the students in each group. The advantage of discrete choice in this particular application is that, as these FoP labels exist in order to influence consumer choice, discrete choice within a diad is a direct measure of their effectiveness [54]. 

**Figure 7:**
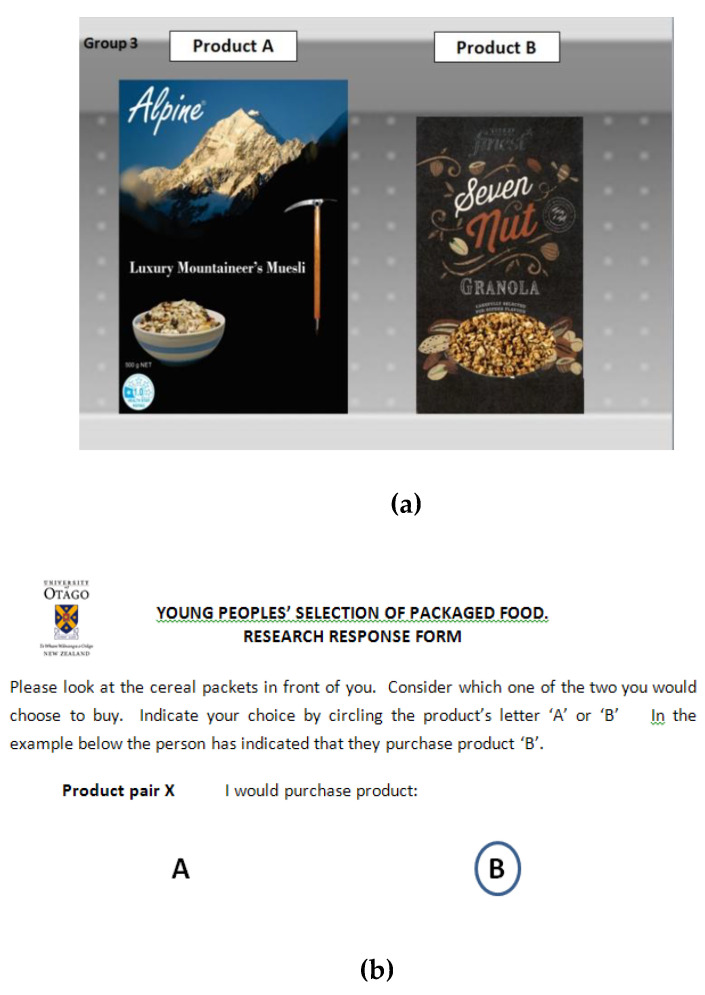
Experimental administration details: (**a**) large scale reproduction of a single-choice diad as shown to each class by on a 1.7 × 1.2 screen. The real products were held up at the same time. (**b**) The response instrument used. In this case, it is the example used to brief each class.

The subjects’ choice of product within each diad was individually self-recorded using the response instrument shown in Figure 7. The choice for each product was coded as ‘1’ if product A was chosen and as ‘0’ if product B was chosen. As the participants were adolescents, who are generally not yet fully sovereign food consumers, this created an issue as to what the decision task should be—purchase or consumption. Pre-administration tests indicated that there was no issue with processing a purchase decision, so this dependent variable was used.

The dependent variable for the analysis was derived from the raw data as the average of the responses for all students in that group, a figure between 0 and 1. So, if out of a sample of 60 students, 40 chose product ‘A’, the raw data point would be a single figure (0.667). As long as the number of responses in each group exceeded thirty, then the requirements of the central limit theory were satisfied, and the averages could be treated as parametric statistics that could be used as an input to an analysis of variance [6,59]. The numbers of participants for each of the four groups were 1: 68, 2: 55, 3: 51, 4: 57.

The use of adolescents for this research did create some challenges. The research was administered in the registration period for each class between 8.40 and 9.00 am. The time available to set the research up, brief the respondents, acquire consents and then acquire responses from up to 25 respondents and vacate the area was typically around 10 minutes. This could be achieved for the three diads within the time allowed, but time constraints precluded any other form of data acquisition beyond very basic demographics. It is for this reason also that each diad was presented to the student groups using a large screen projection in the manner shown in Figure 7. The left-hand product of the diad on the screen was labelled ‘A” and the right-hand product was labelled ‘B’. The real products for each diad were held up at the same time. The screen and real product diads were each visible for a period of 10 seconds (around the time taken to make an FMCG purchase). The three diads shown to each group where shown in succession over a total period of some three minutes. Sixteen classes were surveyed over the research, with four classes randomly assigned to each of the four groups. 

The data were then statistically analyzed using an adjusted analysis of variance [55] with Tukey’s post hoc test for the significance of multiple means [60]

## 3. Results

**Figure 8:**
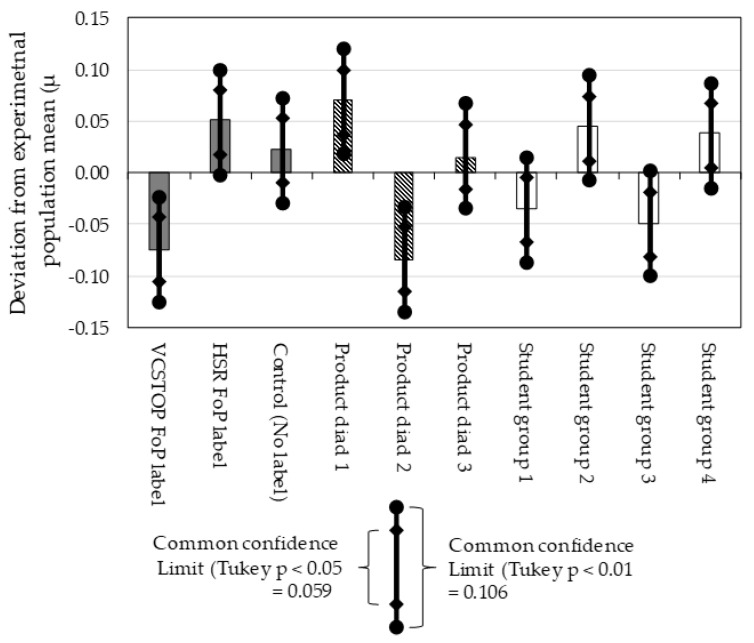
Deviation of FoP treatments (shaded bars), choice diads (hatched bars) and student groups (white bars) from the experimental population mean (*μ* = 0.50). The experimental population mean is the average of the responses from all cells within the experiment.

The results of this research are shown in the form of a bar chart (Figure 8), an adjusted analysis of variance table (Table 1), and a set of significance of multiple means tables (Table 2). The dataset is available as a Supplementary Materials.

The results show a significant interaction in the analysis of variance, but this was not as large as the main effects of the diads and cue treatments. As the Latin square is a fully confounded design, the partial replication test for interaction gives an estimate for interaction that incorporates all possible interactions between the observed levels of the three independent variables, but it does not partition them. The fully confounded nature of the Latin square design means that no definite conclusions can be drawn as to the pattern of interaction, and which independent variables are involved, either by statistical means or by drawing interaction plots.

When an interaction occurs in a Latin square design, the effect is invariably to suppress the statistical significance of the main effects of all the independent variables [6,55,59]. With a partially replicated design, where error is estimated directly via the partial replication, the effect of interaction on the statistics is more nuanced as it is possible to generate results that show both significant main effects and significant interactions. Such a result precludes the drawing of absolute conclusions with regard to the nature and size of the main effects involved, but this does not mean that the result is unusable. 

When this fractionally replicated experimental design was first used by the lead author, research with controlled datasets indicated that as long as interaction accounts for less than 20% of the variance in a design of this type, then interaction derived distortions are at a level that allows the researcher to make indicative conclusions with regard to the size and extent of any main effects with a high (c. 95%) chance of being correct [62]. As the total variance for non-additivity in the analysis of variance table amounts to just under 14% of the total, the results with regard to main effects will be reported and discussed with this caveat in mind. 

The analysis of variance table shows *p* < 0.01 for the main effects of the cue treatments, diads and also for non-additivity (interaction). The impact of student groups was just above *p* < 0.05 in the analysis of variance, but is present at just below *p* < 0.05 in the comparison of multiple means tables. 

The bar chart and the comparison of multiple means tables show that the difference between the control condition, and the Health Star Rating FoP label was not significant, but that the difference between the control and the VCSTOP was significant at *p* < 0.01, while the difference between the two FoP labels was significant at the *p* < 0.05 level. The result of this research is therefore that the VCSTOP label significantly influenced purchase intention by suppressing purchase intention when it was present. This outcome supports Hypothesis 1. The results further indicate that the VCSTOP outperformed the HSR label. However, the presence of interaction within the data rules out a definitive conclusion that it did so to a statistically significant degree. Thus, the results offer qualified support to Hypothesis 2.

## 4. Discussion 

The results of this research support those of Goodman et al. [30] reported in this journal in 2018. Goodman et al.’s research showed that a consumer sample in Canada believed that the VCSTOP based FoP label offered a higher capacity to be noticed and processed than the alternative designs used in the research. This research showed that a VCSTOP based label significantly reduced intent to purchase a range of low nutritional value breakfast cereals that carried the label (Figure 3).

Both results suggest that if the research and policy focus on FoP nutritional labels is moving strongly towards binary warning label systems, then the VCSTOP design, or some derivative of it, should continue to be a serious candidate for consideration as a basis for communicating with consumers via the package at the point of sale. 

At an indicative level, the results also support the results published in this journal in 2016 and 2018 that the Australasian Health Star Rating label is not clearly able to support consumer discrimination against low nutritional value products [5,38]. A definitive conclusion as to the status of the HSR remains elusive. Jones et al. (2019) noted: *“Consumers liked, could understand and use the HSR logo, though effects on purchasing were largely unknown.”* [2]. Bringing some certainty to this status with regard to the cardinal variable of its impact on consumer choice remains a research priority. 

This research raises a number of interesting possibilities for the development of FoP label systems within Australasia and elsewhere. While the HSR is now deployed as an overall evaluation of nutritional value, the governments in Australia and New Zealand are considering the deployment of additional warning labels, specifically for sugar. In a recent policy discussion document, the possible formats for such a system were outlined:

“*There are two potential approaches for this option: Shape or symbol: Use of a particular shape or symbol (e.g., stop sign, give way sign, arrows, exclamation mark) accompanied with text such as ‘high in added sugars’ which would be required for foods that have an added sugars content that exceeds a certain threshold. Text box: A warning text box with a specific message…*”

[63]

It is interesting to note that every one of the suggested symbols is a derivative of a Vienna Convention road sign. This research result and potential future developments of this research have clear implications for this policy initiative.

The primary limitation of this research relates to the presence of interaction in the results. The presence of a significant interaction does mean that results can only be reported at an indicative level. However, in the case of the primary finding above, this can be done with a probability of being correct that corresponds to statistical significance due to the use of a partial replication in the design. The presence of interaction in results generated by this research team is unusual, as considerable effort is taken to avoid it, and these efforts are usually effective. Interaction has not been observed in previous FoP research exercises that have used similar methods, although research using other methodologies has produced indications of it [64,65]. The presence of interaction may be due to a field methodological feature of this school-based research, or it may be more fundamental to the labels, products and diads themselves. 

Until the cause and nature of this interaction can be identified, the use of full-factorial experimental designs, which can deal with non-additivity more effectively than the balanced fractional factorial design used in this research, is strongly recommended to researchers who wish to investigate further in this area. If non-additivity is a basic feature of this research environment, then researchers who use non-replicated fractional factorial designs, such as the Latin square, conjoint analysis, or some computer-driven choice methodologies run a significant risk of either an inconclusive result or an insignificant result that is actually a Type II error.

The research also has limitations due to its extremely narrow focus. (A narrow focus is a prerequisite for both internal and external validity when using experimental instruments of this type). As variations in diet, obesity and behavior towards food have been observed in earlier research [66], the researchers intend to replicate and extend this research into other groups and product environments, but with a full factorial design as the experimental basis. 

The VCSTOP design, incorporating a red octagon with a white border and the word ‘STOP’, does seem to offer obvious advantages as a basis for an FoP warning label system in that it is free to use, standardized across many nations, is ubiquitous and has been consistently deployed in a compatible role within those nations for decades. This creates a level of awareness and consistent interpretation for this cue within the food-consuming public of nearly all nations that is probably beyond the capacity of even the most powerful commercial food brand marks. 

Perhaps the greatest advantage of the pre-existing consumer awareness of the VCSTOP road sign from a research point of view is that its effectiveness as an FoP nutritional warning label aiming to influence consumer choice can be tested before the design is deployed and an investment in supporting it is made. This cannot be done for any nutritional warning design that has no basis of consumer awareness in its target market—no matter what ‘potential’ is suggested for that label by developmental consumer research. 

Given this, it is somewhat puzzling that it has not featured as a basis for any warning label initiatives to date, even though its ordinal traffic light cousin has been deployed in a similar role for decades. Thus, future research should aim to ‘join the dots’ of this research and that of others engaged in warning label research in order to establish whether the VCSTOP is indeed a promising platform for any future warning label system.

## Figures and Tables

**Table 1:** Adjusted analysis of variance table after Youden and Hunter [55].

**			f 0.01,2,11	7.21	
*			f0.05,2,11	3.98	
NS					
	**Sum of Squares**	**Mean Squares**	**Df**	**F**	**Sig.**
Total	0.1001		11		
Consumer group	0.0027	0.0013	2	2.58	NS
Diad	0.0588	0.0294	2	57.16	**
Cue treatment	0.0257	0.0128	2	24.96	**
Interaction	0.0120	0.0060	2	11.64	**
Error	0.0010	0.0003	3		

* Significant at the 1% level, ** Significant at the 5% level, NS Not significant.

**Table 2:** Significance of difference between multiple means table (after Tukey).

Avg. cue trt.1	0.43		Cue Treatment	Cue	Cue	
Avg. cue trt. 2	0.55		Average diff.	trt. 1	trt. 2	
Avg. cue trt 3	0.52		Cue trt. 2	0.13		
Avg. Diad 1	0.57		Cue trt 3	0.10	0.03	
Avg. Diad 2	0.41					
Avg. Diad 4	0.51		Significance	Cue	Cue	
Avg. cons. grp. 1	0.47		Tukey	trt. 1	trt. 2	
Avg. cons. grp. 2	0.55		Cue trt. 2	**		
Avg. cons. grp. 3	0.45		Cue trt. 3	*	NS	
Avg. cons. grp. 4	0.54					
Cue vehicle	Diad	Diad	Cons. Group.	Cons.	Cons.	Cons
avg. diff.	1	2	Avg. diff.	group 1	group 2	group 3
Diad 2	0.16		Cons. group 2	0.08		
Diad 3	0.06	0.16	Cons. group 3	0.02	0.09	
			Cons. Group 4	0.07	0.01	0.09
Significance	Diad	Diad	Significance	Cons.	Cons.	Cons.
(Tukey)	1	2	(Tukey)	group 1	group 2	group 3
Diad 2	**		Cons. group 2	*		
Diad 3	NS	**	Cons. group 3	NS	*	
			Cons. group 4	*	NS	*

* Significant at the 1% level, ** Significant at the 5% level, NS Not significant.

## Supplementary Materials

The following are available online at https://www.mdpi.com/2072-6643/12/6/1545/s1, Dataset.
